# A modified stapled hemorrhoidectomy technique to optimize mucosectomy specimen and improve outcomes

**DOI:** 10.1590/1516-3180.2023.0435.R1.29112024

**Published:** 2025-08-11

**Authors:** Fábio Guilherme Campos, Paula Gabriela Melo Moraes, Pablo Veloso Martins, Leonardo Alfonso Bustamante-Lopez, Carlos Augusto Real Martinez

**Affiliations:** I Department of Surgery, Medical School, Universidade de São Paulo (USP), São Paulo (SP), Brazil.; II Hospital das Clínicas, Medical School, Universidade de São Paulo (USP), São Paulo (SP), Brazil.; III Hospital das Clínicas, Medical School, Universidade de São Paulo (USP), São Paulo (SP), Brazil.; IV Hospital das Clínicas, Medical School, Universidade de São Paulo (USP), São Paulo (SP), Brazil.; V Universidade São Francisco (USF), Bragança Paulista (SP), Brazil.

**Keywords:** Hemorrhoids., Hemorrhoidectomy., Recurrence., Rectal prolapse., Treatment outcome., Complications [subheading]., Surgical choice., Mechanical hemorrhoidopexy., Technical optimization.

## Abstract

**BACKGROUND:**

Surgical treatment of hemorrhoidal disease has undergone numerous modifications in recent decades. Among the technical options, stapled hemorrhoidopexy is currently considered an optimal alternative because it provides a less painful recovery. However, many reports have associated this technique with higher recurrence rates than excisional techniques.

**OBJECTIVES:**

This manuscript presents a technical modification that aims to provide more extensive mucosectomy with mechanical hemorrhoidopexy.

**DESIGN AND SETTING:**

The present technical modification was developed and has been recently used in two hospitals in São Paulo (SP), Brazil.

**METHODS:**

To achieve this, we placed a circumferential submucosal suture at the 3 o’clock position in the clockwise direction. When the left lateral position (9 o’clock) was reached, a loop of 2-0 non-absorbable suture thread was passed around the continuous suture and retracted to the left. Subsequently, the original suture progressed towards the point on the right lateral side, where it was started.

**RESULTS:**

Specifically, the modification consists of establishing two traction points from the pursestring suture; thus, the rectal mucosa entering the stapler head will be more uniform, and the retrieved mucosal strip will present a greater height. These features may play a role in effectively reducing mucosal prolapse and alleviating the symptoms.

**CONCLUSIONS:**

The proposed modification of the original operative technique is simple and aims to improve postoperative results by increasing the height of the mucosal specimen to be resected, thereby reducing long-term recurrence. In the future, this hypothesis will be tested in a randomized study comparing the mucosectomy height and postoperative outcomes of both technical options (classical and present).

## INTRODUCTION

 Hemorrhoidal disease (HD) affects a large portion of the population and may significantly impact quality of life. While many patients benefit from clinical guidance on managing constipation, improving hygiene, and modifying personal habits, a substantial group still requires procedures to control symptoms^1^ . 

 Hemorrhoid excision techniques remain the gold standard for HD management. Ongoing discussions about the optimal surgical approach have focused on early postoperative outcomes such as bleeding, wound healing, and pain, particularly in comparative studies of the Milligan-Morgan and Ferguson techniques.^2^

 The introduction of non-excisional procedures such as Stapled Hemorrhoidectomy (SH) and Doppler-Guided Hemorrhoidal Dearterialization with mucopexy (DG-HAL) has offered patients with grade III and IV disease an alternative that often allows a more comfortable recovery. As a result, attention has shifted toward evaluating long-term outcomes, especially symptom persistence or recurrence.^3^

 Stapled Hemorrhoidectomy (also known as stapled hemorrhoidopexy or mechanical anopexy), introduced by Longo in 1998,^4^ uses a circular stapler to remove a circumferential strip of rectal mucosa. This procedure aims to reduce prolapse by repositioning the hemorrhoidal cushions to their original anatomical location, based on the hypothesis that internal rectal prolapse contributes to disease development.^5^

 By correcting the prolapse, the stapler-induced mucosectomy may also reduce submucosal blood flow to the hemorrhoidal plexus while preserving the anoderm from surgical trauma. This technique seeks to relieve symptoms while maintaining normal anatomy and physiological function. Today, SH is considered a major and innovative advancement in HD treatment.^6^

## OBJECTIVE

 The present study aims to present this idea and discuss the supposed advantages of a technical modification to classical mucosectomy provided by mechanical staplers. 

## METHODS

 Our routine preparation involved an in-hospital procedure performed under sedation and spinal anesthesia. Preoperative measures included a rectal washout and intravenous antibiotics administered one hour before surgery. With the patient in the lithotomy and Trendelenburg positions, internal anorectal prolapse was assessed through digital examination and by inserting and retrieving endoanal gauze, applying inward traction during the maneuver. 

 A Circular Anal Dilator (CAD33, Ethicon Endo-Surgery, Raritan, New Jersey, United States) was inserted endoanally and secured to the perianal skin with stay sutures. A PurseString Suture Anoscope was then introduced to facilitate the creation of a submucosal continuous purse-string suture using 2-0 Prolene. 

## RESULTS

**Figure 1:**
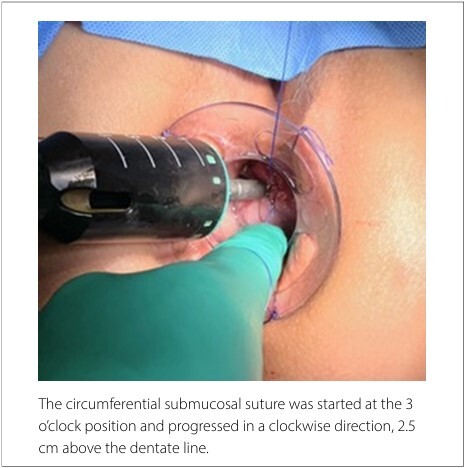
Start of the circumferential suture.

**Figure 2:**
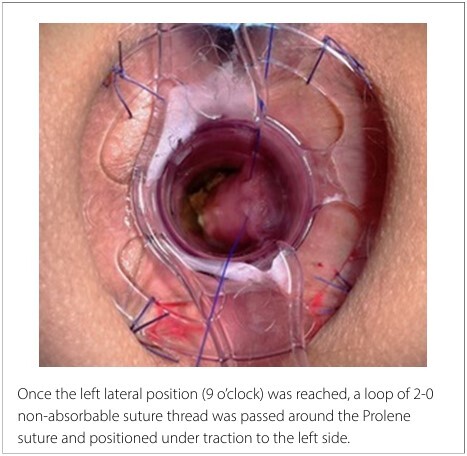
Technical modification.

 The proposed modification involves starting the circumferential submucosal suture at the 3 o’clock position and progressing in a clockwise direction (Figure 1). When the left lateral position (9 o’clock) was reached, a loop of 2-0 non-absorbable suture thread was passed around the Prolene suture and positioned under traction to the left side (Figure 2). Subsequently, the original Prolene suture continued toward the initial point on the right lateral side where it had started. 

**Figure 3:**
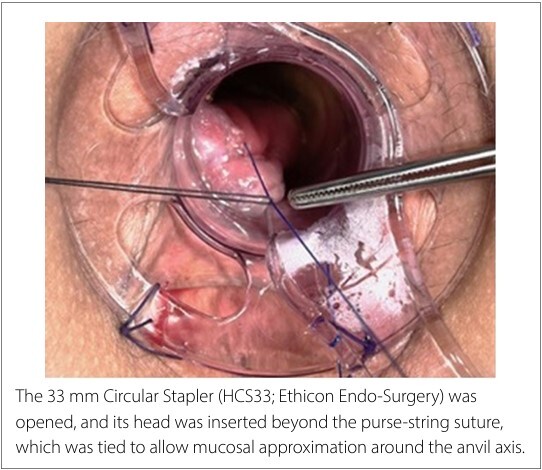
Insertion of the circular stapler.

**Figure 4:**
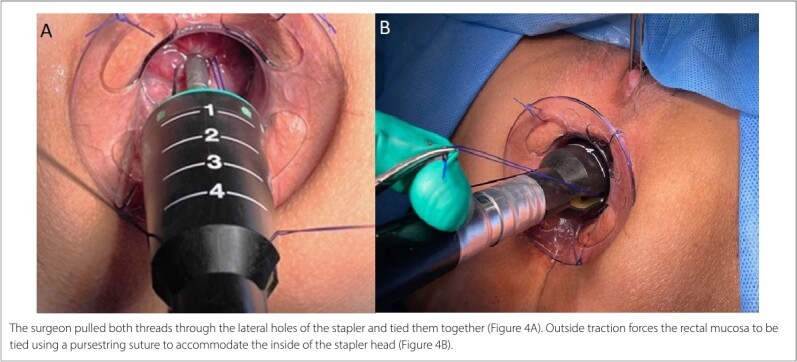
Traction of both threads.

**Figure 5:**
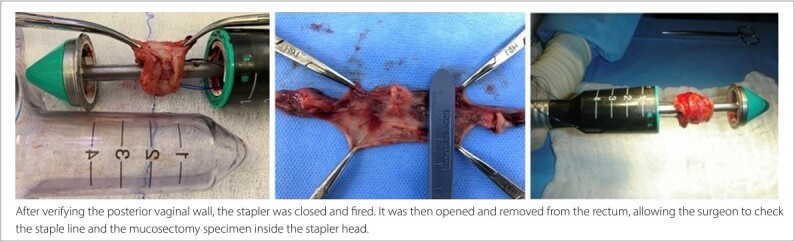
Opening of the stapler to retrieve the mucosectomy specimen.

 At this point, two traction points were identified in opposite positions: the Prolene suture on the right (3 o’clock) and the secondary thread on the left (9 o’clock). A 33 mm Circular Stapler (HCS33; Ethicon Endo-Surgery, Raritan, New Jersey, United States) was then opened, and its head was introduced beyond the pursestring suture, which was tied to allow mucosal approximation around the anvil axis (Figure 3). The surgeon pulled both threads through the lateral holes of the stapler, guiding the rectal mucosa enclosed by the purse-string suture to fit inside the stapler head (Figures 4A and 4B). After confirming the position of the posterior vaginal wall, the stapler was closed and fired. It was then opened and removed from the rectum, allowing inspection of the staple line and the mucosectomy specimen within the stapler head (Figure 5). 

## DISCUSSION

 A stapled hemorrhoidectomy was performed to minimize postoperative pain. Over the years, this procedure has been increasingly adopted, despite concerns about disease recurrence. As understanding of patient needs has evolved, the technique has been combined with excisional methods to improve outcomes while still reducing pain and discomfort.^7^

 Ongoing debate about long-term results underscores the importance of informing patients about the technical aspects and key considerations involved in this surgical choice. Patients must be fully informed about the benefits, risks, and expected outcomes. 

 Mechanical hemorrhoidectomy is intended to perform a circumferential mucosectomy to restore local anatomy and correct prolapse that occurs during defecation. Careful attention to technical detail is essential for minimizing complications and achieving optimal outcomes. In this context, colorectal surgeons have recognized that the purse-string suture should ideally be placed 2.5-3.5 cm above the dentate line.^8^

 Historically, when Procedure for Prolapse and Hemorrhoids (PPH) was introduced, the initial approach involved placing the suture higher in the rectum. However, this failed to adequately address the external hemorrhoidal components. On the other hand, placing the suture too low risks incorporating the dentate line into the mucosectomy, which can result in postoperative pain and dysfunction. It is now understood that the purse-string suture must not be placed too high (to ensure effective prolapse correction), too deep (to avoid including muscle fibers), or too low (to prevent stapling near the sensitive dentate line).^7^

 The technique described here represents a modification designed to optimize the mucosectomy achieved with the stapler. In the classical approach, both threads are pulled from the 12 o’clock position, meaning traction is applied from a single (anterior) point. This can result in uneven mucosal entry into the stapler head. By contrast, applying suture traction from two opposite points is more likely to produce a regular, symmetric mucosal strip and increase the amount of tissue drawn into the stapler. 

**Figure 6:**
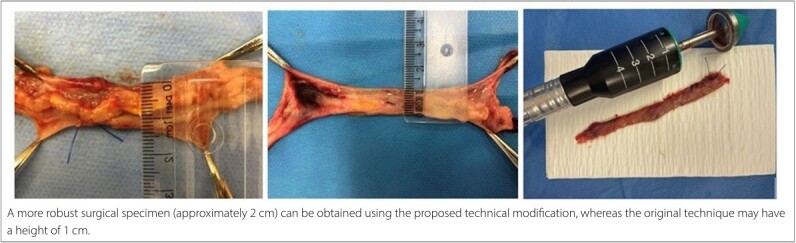
A better specimen is observed.

 This modified method often results in a more substantial surgical specimen, as indicated by its gross appearance and height. A high-quality specimen may measure around 2 cm in height, compared to the roughly 1 cm height typical of specimens from the classical technique. Figure 6 illustrates specimens obtained using both methods. 

## CONCLUSIONS

 The proposed modification of the original operative technique is simple and aims to improve postoperative results by increasing the height of the mucosal specimen to be resected, thereby reducing long-term recurrence. In the future, this hypothesis will be tested in a randomized study comparing the mucosectomy height and postoperative outcomes of both technical options (classical and present). 
